# Association between urinary uric acid excretion and kidney outcome in patients with CKD

**DOI:** 10.1038/s41598-024-55809-9

**Published:** 2024-03-01

**Authors:** Yuta Asahina, Yusuke Sakaguchi, Tatsufumi Oka, Koki Hattori, Takayuki Kawaoka, Yohei Doi, Ryohei Yamamoto, Isao Matsui, Masayuki Mizui, Jun-Ya Kaimori, Yoshitaka Isaka

**Affiliations:** 1 Department of Nephrology, Osaka University Graduate School of Medicine, Suita, Japan; 2 Health and Counseling Center, Osaka University, Toyonaka, Japan

**Keywords:** Medical research, Nephrology

## Abstract

Inhibiting tubular urate reabsorption may protect the kidney from urate-induced tubular injury. However, this approach may promote intratubular uric acid crystallization, especially in acidified urine, which could be toxic to the kidney. To assess how tubular urate handling affects kidney outcomes, we conducted a retrospective cohort study including 1042 patients with estimated glomerular filtration rates (eGFR) of 15–60 mL/min/1.73 m^2^. The exposures were fractional excretion of uric acid (FEUA) and urinary uric acid-to-creatinine ratio (UUCR). The kidney outcome was defined as a halving of eGFR from baseline or initiating kidney replacement therapy. The median FEUA and UUCR were 7.2% and 0.33 g/gCre, respectively. During a median follow-up of 1.9 years, 314 kidney outcomes occurred. In a multivariate Cox model, the lowest FEUA quartile exhibited a 1.68-fold higher rate of kidney outcome than the highest FEUA quartile (95% confidence interval, 1.13–2.50; P = 0.01). Similarly, lower UUCR was associated with a higher rate of kidney outcome. Notably, patients in the highest quartile of FEUA and UUCR were at the lowest risk of kidney outcome even among those with aciduria. In conclusion, lower FEUA and UUCR were associated with a higher risk of kidney failure, suggesting that increased urate reabsorption is harmful to the kidney.

## Introduction

Hyperuricemia is highly prevalent among patients with chronic kidney disease (CKD)^1^. Over the last decades, there has been a controversy as to whether hyperuricemia is causally related to the progression of CKD or merely a risk marker reflecting deteriorated kidney function^2,3^. Cohort studies showed that hyperuricemia was associated with the incidence and progression of CKD^4–10^, albeit not consistently^11,12^. However, a Mendelian randomization study did not prove a causal link between serum urate levels and the risk of CKD^13^. Furthermore, large-scale randomized controlled trials failed to show a benefit of allopurinol on kidney outcomes although they included patients with normal serum urate levels^14,15^. Thus, lowering serum urate levels per se may not improve kidney prognosis.

Uric acid load to the kidney, rather than hyperuricemia, may be more directly involved in kidney injury^16,17^. For example, a cohort study has reported that hyperuricemia was not associated with a faster decline in kidney function except in individuals with impaired function of ATP-binding cassette subfamily G member 2 (ABCG2), a dominant transporter of intestinal urate excretion, which leads to a compensatory increase in uric acid excretion from the kidney^18^. Mechanistically, basic studies have suggested that urate reabsorbed in the proximal tubules accelerates kidney damage through inducing oxidative stress, inflammation, cell death, and lysosomal damage^3,19–21^. Notably, inhibition of URAT1, a major transporter for urate reabsorption, prevents urate-induced tubular cell injury^22,23^. This is consistent with a clinical finding that plasma urate-lowering effect of sodium–glucose cotransporter 2 (SGLT2) inhibitors via suppressing urate reabsorption partly mediated an improvement in kidney outcomes by these drugs^24,25^.

It is concerned, however, that inhibiting urate reabsorption elevates intratubular urate concentrations, predisposing uric acid crystallization especially in acidified urine, which could be toxic to the kidney^26^. It has not been well studied how the tubular handling of urate affects the prognosis of CKD. Here, we examined fractional excretion of uric acid (FEUA) and a urinary uric acid-to-creatinine ratio (UUCR), and their associations with kidney outcomes in patients with CKD.

## Methods

### Study population

This retrospective cohort study included patients who were referred to the Department of Nephrology at Osaka University Hospital from January 2005 to March 2022. We included patients (1) who were aged 20 years or older; (2) whose baseline estimated glomerular filtration rate (eGFR) was 15‒60 mL/min/1.73 m^2^; and (3) who had never received kidney replacement therapy (KRT). Patients were excluded if they had hypouricemia (baseline serum uric acid levels of < 119 μmol/L). Patients were followed up from the day of the first available data on FEUA until the occurrence of kidney outcome, death, lost to follow-up, or the end of the study period (March 31, 2022), whichever came first. The study protocol was approved by the Ethics Committee of Osaka University Hospital (Approval Number 21513). The requirement for informed consent was waived due to the retrospective nature of the study design and anonymous clinical data. This study was conducted in accordance with the principles of the Declaration of Helsinki.

### Outcome

The study outcome was a kidney composite endpoint defined as a ≥ 50% decline in eGFR from baseline values or initiation of KRT (chronic dialysis or kidney transplantation). We assessed death as a competing risk event. The dates of the initiation of KRT and death were ascertained through a review of patients’ medical records.

### Exposures

The main exposure was baseline FEUA. FEUA was calculated as 100 × ([urine uric acid [UUA] (mg/dL)] × [serum creatinine (mg/dL)]) / ([urine creatinine (mg/dL)] × [serum urate (mg/dL)]). We additionally examined UUCR ([urine uric acid (mg/dL)]/[urine creatinine (mg/dL)]) as a surrogate of the amount of urinary uric acid excretion. These indices were calculated using measurements from spot urine samples. FEUA and UUCR were categorized into quartiles. During a median follow-up of 1.9 (interquartile range, 0.5‒4.8) years, FEUA and UUCR were measured 1.8 times (standard deviation [SD] 2.4) on average.

### Data collection and measurements

Demographics and comorbidities were collected from the electronic data capture system integrated in the electronic medical records of Osaka University Hospital, which can automatically extract individual patients’ medical data. These data included age, sex, body mass index (BMI), blood pressure, diabetes mellitus, history of gout, and cardiovascular comorbidities (coronary artery diseases [angina pectoris and myocardial infarction] requiring percutaneous coronary intervention and/or coronary artery bypass graft, congestive heart failure, valvular heart diseases, and stroke [cerebral infarction and intracranial hemorrhage]). Laboratory data included serum albumin, serum creatinine, hemoglobin, potassium, phosphate, serum urate, C-reactive protein (CRP), urine pH, urinary protein-to-creatinine ratio (UPCR), urine creatinine, and UUA. Prescription data included loop and thiazide diuretics, angiotensin-converting enzyme inhibitors (ACEIs), angiotensin II receptor blockers (ARBs), xanthine oxidase inhibitors, uricosuric agents, and sodium–glucose cotransporter 2 inhibitors. These data were collected throughout the study period and treated as time-dependent variables in marginal structural models (MSM).

The eGFR was calculated using the following equation for the Japanese^27^:

$$ {\text{eGFR}}\;({\text{mL/min/}}1.73 \;{\text{m}}^{2} ) = 194 \times {\text{serum}}\;{\text{creatinine}}^{ - 1.094} \times {\text{age}}^{ - 0.287} \;( \times \,0.739\;{\text{if}}\;{\text{female}}). $$

### Statistical analysis

Baseline data were summarized as number (percent) for categorical variables or as mean (SD) for continuous variables with normal distribution or median (interquartile range) for variables with skewed distribution. Baseline characteristics were compared across quartiles of FEUA and UUCR using Cuzick’s test for trend for continuous variables^28^ or Mantel–Haenszel test for trend for categorical variables. A relationship between FEUA and UUCR was depicted using a restricted cubic spline curve with 3 knots (10th, 50th, and 90th percentiles of UUCR).

A Cox proportional hazards model was used to estimate hazard ratio (HR) and 95% confidence intervals (CI) for kidney outcome. Multivariable Cox models were adjusted for the following baseline covariates: age, sex, BMI, systolic blood pressure, diabetes mellitus, cardiovascular comorbidities, eGFR, hemoglobin, potassium, phosphate, serum urate, CRP, UPCR, diuretics, ACEIs/ARBs, and xanthine oxidase inhibitors. For covariates that violated the proportional hazards assumption (i.e., albumin), the corresponding time interaction term was included in the models. We calculated E-values to estimate the magnitude of unmeasured confounders that are required to overcome the observed association between the exposures and outcome^29,30^. Restricted cubic spline curves with three knots (10th, 50th, and 90th percentiles of FEUA and UUCR) were drawn to illustrate a non-linear relationship between each exposure as a continuous variable and the hazard of the outcome. Effect modification was evaluated by incorporating an interaction term between the exposures and baseline covariates including age, sex, diabetes mellitus, cardiovascular comorbidities, serum urate levels (≥ 416, < 416 μmol/L), urine pH (< 6.0, ≥ 6.0), UPCR (≥ 0.5, < 0.5 g/gCre), eGFR (≥ 30, < 30 mL/min/1.73 m^2^), diuretics, and xanthine oxidase inhibitors. Furthermore, given a potential importance of aciduria (urine pH < 6.0) in the interpretation of our findings, we performed a subgroup analysis based on urine pH.

We performed several sensitivity analyses. First, we used the Fine and Gray method to account for death as a competing-risk event. Second, we repeated the Cox model after excluding (1) those who were followed up for less than 90-days, and (2) those who had a history of gout. Finally, in order to account for time-dependent confounding, we employed marginal structural models (MSM) to estimate the association between the time-updated exposures and kidney outcome^31,32^. In this study, eGFR was considered the main time-dependent confounder because eGFR could affect both exposures (FEUA and UUCR) and the outcome (kidney failure), whereas eGFR might be influenced by previous exposures (FEUA and UUCR). The cutoff points of FEUA (< 4.8 vs ≥ 4.8%) and UUCR (< 0.22 vs ≥ 0.22 mg/gCre) were chosen based on the bottom 25th percentile of each variable. The detailed method of MSM is described in Supplemental methods.

As an additional analysis, we examined an association between UUA and the outcome since the concentration of uric acid in the urine might be more directly involved in the formation of uric acid crystals than FEUA or UUCR. This analysis was performed by a baseline Cox proportional hazards model including all covariates used in the analyses for FEUA and UUCR, with a stratification by urine pH.

Missing data at baseline, except for FEUA and UUCR, were imputed using multiple imputation by chained equation (MICE) which included all baseline covariates. Continuous variables with missing data (BMI, systolic blood pressure, hemoglobin, potassium, phosphate, albumin, CRP, UPCR) were imputed based on linear regression imputation including all baseline covariates. We created five imputed datasets, which were analyzed separately and combined using Rubin’s rules. Missing data during the follow-up period were imputed by the last observation carried forward method.

Statical analyses were performed using STATA/SE, version 16 (STATA Corp, College Station, TX).

## Results

### Baseline characteristics

Among 3794 patients who met the inclusion and exclusion criteria, 1042 (27%) had available data on FEUA (Fig. S1). Demographic and clinical characteristics were similar between those with and without data on FEUA (Table S1).

**Figure 1:**
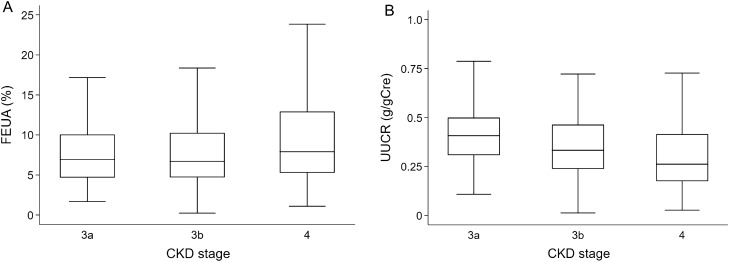
FEUA and UUCR across CKD stages. Box plots show that patients in higher CKD stages have (**A**) higher FEUA and (**B**) lower UUCR. *FEUA* fractional excretion of uric acid, *UUCR* urinary uric acid-to-creatinine ratio, *CKD* chronic kidney disease.

The mean (SD) baseline eGFR of 1042 study patients was 35 (12) mL/min/1.73 m^2^. The median [interquartile range] values of FEUA and UUCR were 7.2 [4.8‒10.9] % and 0.33 [0.22‒0.47] g/gCre, respectively. Patients in higher CKD stages had higher FEUA (stage 3a, 6.9 [4.7–10.1] %; stage 3b, 6.7 [4.7–10.3] %; stage 4, 7.9 [5.3–12.9] %; P < 0.001 by Kruskal–Wallis test) but lower UUCR (stage 3a, 0.41 [0.31–0.50] g/gCre; stage 3b, 0.33 [0.24–0.46] g/gCre; stage 4: 0.26 [0.18–0.42] g/gCre; P < 0.001 by Kruskal–Wallis test) (Fig. 1).

Baseline characteristics stratified by FEUA quartile are summarized in Table 1. Patients in the lower FEUA quartile were more likely to be male and had a higher prevalence of cardiovascular comorbidities and gout. They also showed lower UPCR, higher serum urate levels, and were more likely to have aciduria. In addition, they received diuretics and xanthine oxidase inhibitors more frequently. Baseline characteristics stratified by UUCR quartile are summarized in Table S2.

**Table 1:** Baseline characteristics according to FEUA quartile.

Characteristics	Total	Missing data	FEUA quartile (%)				P for trend
			Q1: < 4.8	Q2: 4.8–7.2	Q3: 7.2–10.9	Q4: > 10.9	
	n = 1042	n (%)	n = 261	n = 256	n = 264	n = 261	
Age, year	63 (16)	0	63 (17)	64 (15)	64 (15)	61 (15)	0.2
Male	667 (64%)	0	192 (74%)	165 (64%)	159 (60%)	151 (58%)	< 0.001
BMI, kg/m^2^	23.3 (4.3)	146 (14%)	23.5 (4.6)	23.7 (4.3)	23.1 (4.2)	22.8 (4.4)	0.02
SBP, mmHg	130 (23)	194 (19%)	125 (23)	129 (21)	133 (23)	133 (25)	< 0.001
DBP, mmHg	75 (15)	194 (19%)	72 (15)	76 (15)	75 (14)	77 (15)	< 0.001
Diabetes mellitus	433 (42%)	0	108 (41%)	100 (39%)	116 (44%)	109 (42%)	0.7
Cardiovascular comorbidities	251 (24%)	0	84 (32%)	57 (22%)	61 (23%)	49 (19%)	0.001
Gout	84 (8%)	0	35 (13%)	19 (7%)	21 (8%)	9 (3%)	< 0.001
ACEIs/ARBs	481 (46%)	0	146 (56%)	132 (52%)	106 (40%)	97 (37%)	< 0.001
Loop diuretics	458 (44%)	0	148 (57%)	96 (38%)	109 (41%)	105 (40%)	0.001
Thiazide diuretics	150 (14%)	0	64 (24%)	42 (16%)	30 (11%)	14 (5%)	< 0.001
Xanthine oxidase inhibitors	345 (33%)	0	144 (55%)	99 (39%)	72 (27%)	30 (11%)	< 0.001
Uricosuric agents	36 (3%)	0	9 (3%)	7 (3%)	9 (3%)	11 (4%)	0.6
SGLT2 inhibitors	20 (2%)	0	5 (2%)	5 (2%)	8 (3%)	2 (1%)	0.5
Hemoglobin, g/dL	11.5 (2.2)	46 (4%)	12.1 (2.2)	12.1 (2.2)	11.4 (2.1)	10.5 (2.1)	< 0.001
Potassium, mEq/L	4.3 (0.6)	11 (1%)	4.3 (0.6)	4.3 (0.5)	4.3 (0.6)	4.2 (0.7)	0.01
Phosphate, mg/dL	3.5 (0.8)	232 (22%)	3.6 (0.7)	3.5 (0.7)	3.5 (0.8)	3.3 (0.9)	0.02
Albumin, g/dL	3.4 (0.8)	47 (4%)	3.6 (0.7)	3.6 (0.7)	3.3 (0.8)	3.2 (0.8)	< 0.001
eGFR, mL/min/1.73 m^2^	35 (12)	0 (0%)	36 (12)	37 (12)	35 (13)	32 (13)	< 0.001
Serum urate, μmol/L	411 (126)	0 (0%)	473 (134)	436 (104)	400 (106)	335 (114)	< 0.001
C-reactive protein, mg/dL	0.2 [0.0–1.0]	94 (9%)	0.1 [0.0–0.6]	0.1 [0.0–0.4]	0.2 [0.0–1.3]	0.4 [0.1–2.4]	< 0.001
Aciduria*	433 (42%)	4 (< 1%)	132 (51%)	117 (46%)	109 (42%)	75 (29%)	< 0.001
UPCR, g/gCre	0.5 [0.1–2.2]	270 (26%)	0.3 [0.0–1.3]	0.4 [0.1–1.9]	0.6 [0.1–2.9]	1.0 [0.4–3.2]	< 0.001
FEUA, %	7.2 [4.8–10.9]	0 (0%)	3.8 [3.0–4.4]	6.0 [5.5–6.6]	8.7 [7.9–9.9]	15.0 [12.6–19.6]	< 0.001
UUCR, g/gCre	0.33 [0.22–0.47]	0 (0%)	0.18 [0.13–0.25]	0.29 [0.24–0.36]	0.38 [0.30–0.48]	0.51 [0.42–0.62]	< 0.001

*Aciduria is defined as urine pH < 6.0.

*BMI* body mass index, *SBP* systolic blood pressure, *DBP* diastolic blood pressure, *ACEIs/ARBs* angiotensin-converting enzyme inhibitors/angiotensin II receptor blockers, *SGLT2* sodium–glucose cotransporter 2, *eGFR* estimated glomerular filtration rate, *UPCR* urinary protein-to-creatinine ratio, *FEUA* fractional excretion of uric acid, *UUCR* urinary uric acid-to-creatinine ratio.

### Correlation between FEUA and UUCR

**Figure 2:**
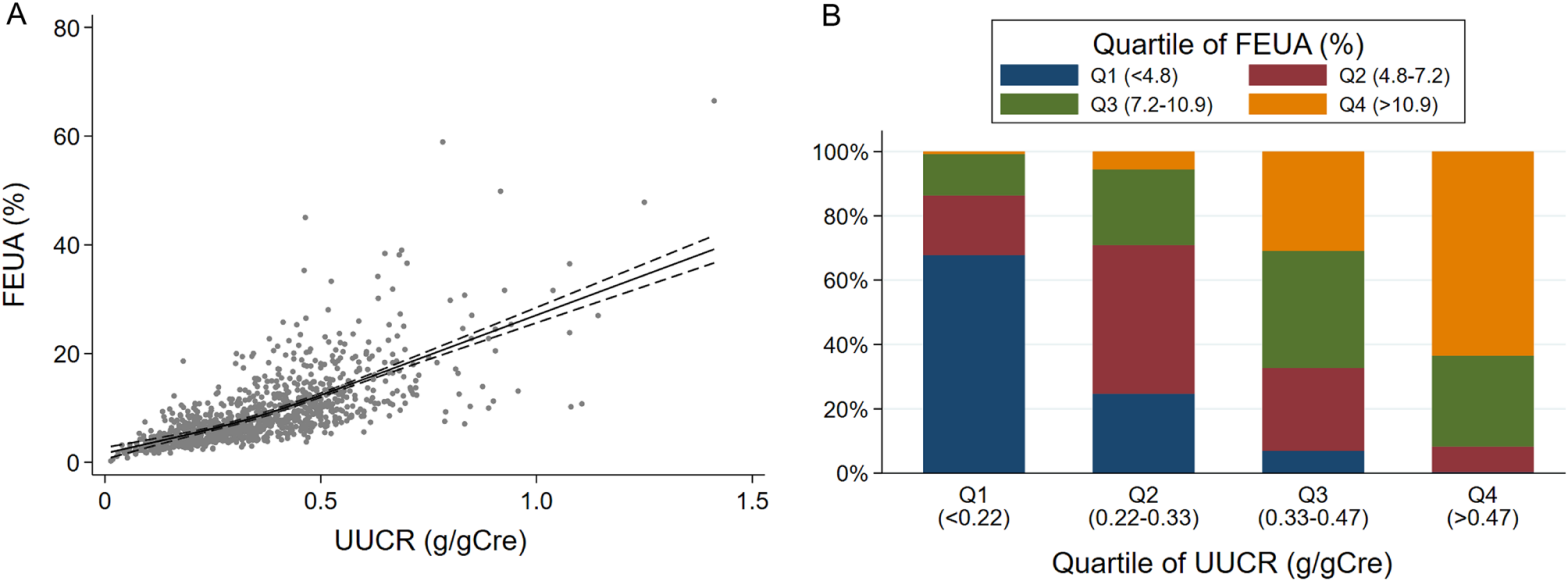
Correlation between FEUA and UUCR. (**A**) A restricted cubic spline curve with three knots (10th, 50th, and 90th percentiles of UUCR) shows an almost linear relationship between UUCR and FEUA. The dashed lines denote 95% confidence intervals. (**B**) Distribution of quartiles of FEUA by quartiles of UUCR. Most of the patients in the lowest UUCR quartile are in the lowest or the second lowest FEUA quartile (68% and 18%, respectively). *FEUA* fractional excretion of uric acid, *UUCR* urinary uric acid-to-creatinine ratio.

There was a strong correlation between FEUA and UUCR (correlation coefficient, 0.71; P < 0.001). A restricted cubic spline curve showed an almost linear relationship between FEUA and UUCR (Fig. 2A). Most of the patients in the lowest UUCR quartile were in the lowest or the second lowest FEUA quartile (68% and 18%, respectively) (Fig. 2B). In a multivariate linear regression model, FEUA was more strongly correlated with UUCR than eGFR (Table 2).

**Table 2:** A multivariate linear regression analysis for the association of FEUA and eGFR with UUCR.

	Standardized β [95% CI]	P-value
FEUA	0.91 [0.87, 0.95]	< 0.001
eGFR	0.53 [0.49, 0.57]	< 0.001

Models are adjusted for age, sex, body mass index, systolic blood pressure, diabetes mellitus, cardiovascular comorbidities, albumin, hemoglobin, potassium, phosphate, serum urate, C-reactive protein, urinary protein-to-creatinine ratio, loop and thiazide diuretics, angiotensin-converting enzyme inhibitors/angiotensin receptor blockers, and xanthine oxidase inhibitors.

Each variable is standardized to a mean of zero and a standard deviation of one.

*FEUA* fractional excretion of uric acid, *UUCR* urinary uric acid-to-creatinine ratio, *eGFR* estimated glomerular filtration rate, *CI* confidence interval.

### Associations of FEUA and UUCR with kidney outcome

**Figure 3:**
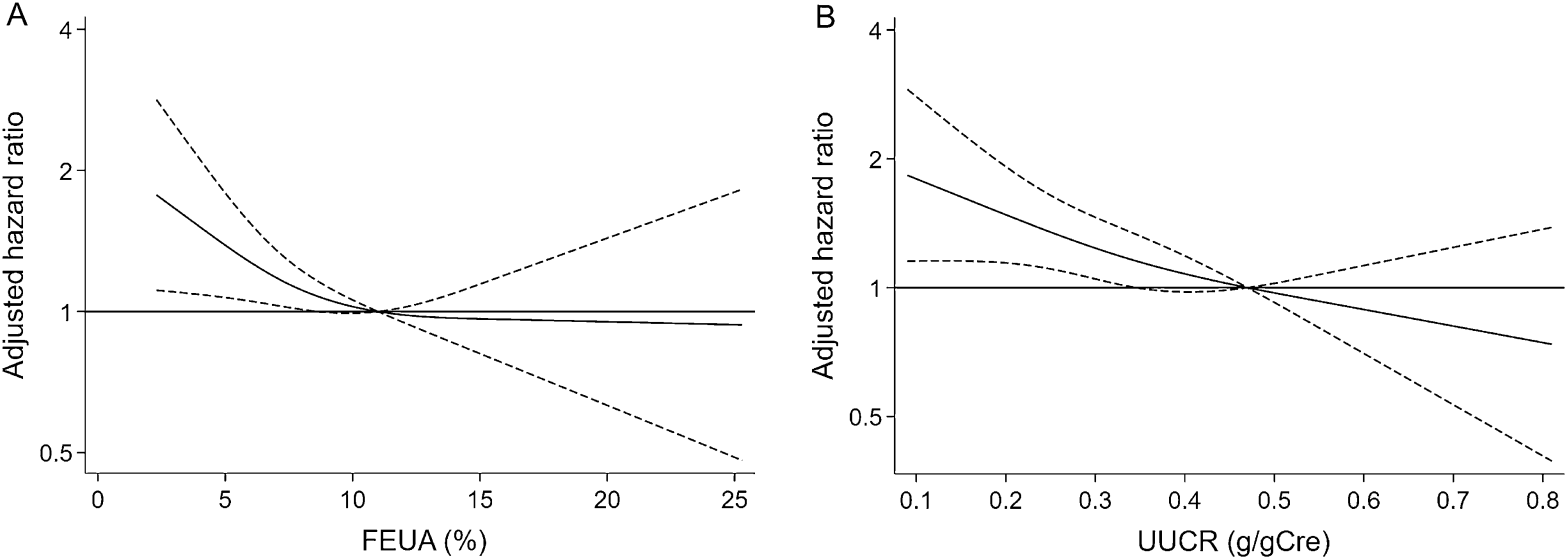
Associations of FEUA and UUCR with kidney outcome. Restricted cubic spline curves show the relationship between adjusted hazard ratio for kidney outcome and (**A**) FEUA or (**B**) UUCR with three knots (10th, 50th, and 90th percentiles of each exposure). Model are adjusted for age, sex, body mass index, systolic blood pressure, diabetes mellitus, cardiovascular comorbidities, estimated glomerular filtration rate, potassium, phosphate, albumin, serum urate, C-reactive protein, urinary protein-creatinine ratio, loop and thiazide diuretics, angiotensin-converting enzyme inhibitors and angiotensin II receptor blockers, and xanthine oxidase inhibitors. The solid lines denote adjusted hazard ratio and the dashed lines denote 95% confidence intervals. The reference lines denote an adjusted hazard ratio of 1.0. *FEUA* fractional excretion of uric acid, *UUCR* urinary uric acid to creatinine ratio.

During a median follow-up of 1.9 [interquartile range, 0.5‒4.8] years, 314 kidney outcomes occurred and 122 died. In the multivariate Cox model, there was a dose-dependent association between FEUA and kidney outcome; those in the lowest FEUA quartile had a 1.68-fold (95% CI, 1.13‒2.50; P = 0.01; E-value = 2.21) higher rate of kidney outcome than those in the highest FEUA quartile (Table 3). A restricted cubic spline curve showed that the adjusted HR increased when FEUA became less than approximately 10% (Fig. 3A). There was no significant effect modification by baseline covariates including age, sex, diabetes mellitus, cardiovascular comorbidities, serum urate levels, urine pH, UUCR, eGFR, UPCR, diuretics, and xanthine oxidase inhibitors. Similar associations were found between UUCR and kidney outcome (Table 3; Fig. 3B).

**Table 3:** Cox proportional hazards models for the associations of FEUA and UUCR with kidney outcome.

Exposures	FEUA quartile (%)				UUCR quartile (g/gCre)			
	Q1: < 4.8 (n = 261)	Q2: 4.8–7.2 (n = 256)	Q3: 7.2–10.9 (n = 264)	Q4: > 10.9 (n = 261)	Q1: < 0.22 (n = 263)	Q2: 0.22–0.33 (n = 251)	Q3: 0.33–0.47 (n = 275)	Q4: > 0.47 (n = 252)
No. of events	86	72	81	75	94	72	79	69
Incidence rate, 100 p-y (95% CI)	10.6 (8.6–13.1)	8.7 (6.9–11.0)	9.5 (7.7–11.8)	9.8 (7.8–12.3)	13.9 (11.4–17.0)	9.2 (7.3–11.6)	7.7 (6.2–9.6)	9.0 (7.1–11.3)
Hazard Ratio (95% CI)	1.68 (1.13–2.50)	1.48 (1.00–2.19)	1.22 (0.87–1.71)	Ref	1.71 (1.17–2.49)	1.14 (0.80–1.63)	1.04 (0.74–1.47)	Ref
P-value	0.01	0.05	0.2	–	0.005	0.5	0.8	–

Models are adjusted for age, sex, body mass index, systolic blood pressure, diabetes mellitus, cardiovascular comorbidities, albumin, estimated glomerular filtration rate, hemoglobin, potassium, phosphate, serum urate, C-reactive protein, urinary protein-to-creatinine ratio, loop and thiazide diuretics, angiotensin-converting enzyme inhibitors/angiotensin receptor blockers, and xanthine oxidase inhibitors.

*FEUA* fractional excretion of uric acid, *UUCR* urinary uric acid-to-creatinine ratio, *p-y* person-years, *CI* confidence interval.

In the subgroup analysis based on urine pH, the highest quartile of FEUA and UUCR showed the lowest rate of kidney outcome even in patients with aciduria (Fig. S2).

### Sensitivity analysis

The results were not substantially altered (1) when death was considered as a competing risk event (Table S3), (2) after excluding those who were followed up for less than 90 days (Table S4), and (3) after excluding those who had a history of gout (Table S4).

The MSM exhibited a similar association between time-updated FEUA and UUCR and kidney outcome. Both lower FEUA (< 4.8%) and UUCR (< 0.22 g/gCre) were associated with a higher rate of kidney outcome (Table 4).

**Table 4:** Marginal structural models for the associations of FEUA and UUCR with kidney outcome.

	Hazard ratio	95% CI	P-value
FEUA			
< 4.8%	1.41	1.01–1.97	0.04
≥ 4.8%	Ref	–	–
UUCR			
< 0.22 g/gCre	1.85	1.31–2.61	< 0.001
≥ 0.22 g/gCre	Ref	–	–

Models are adjusted for baseline covariates including age, sex, body mass index, systolic blood pressure, diabetes mellitus, cardiovascular comorbidities, albumin, estimated glomerular filtration rate, hemoglobin, potassium, phosphate, serum urate, C-reactive protein, urinary protein-to-creatinine ratio, loop and thiazide diuretics, angiotensin-converting enzyme inhibitors/angiotensin receptor blockers, and xanthine oxidase inhibitors, and time-varying covariates including estimated glomerular filtration rate, phosphate, serum urate, urinary protein-to-creatinine ratio, loop and thiazide diuretics, angiotensin-converting enzyme inhibitors/angiotensin receptor blockers, and xanthine oxidase inhibitors.

*FEUA* fractional excretion of uric acid, *UUCR* urinary uric acid-to-creatinine ratio, *CI* confidence interval.

### Additional analysis

Baseline characteristics stratified by UUA quartile are summarized in Table S5. Lower UUA quartiles were associated with a higher rate of the kidney outcome (Table S6). This association was not significantly modified by urine pH (Fig. S2).

## Discussion

Inhibition of tubular urate reabsorption might prevent urate-induced tubular cell injury^3,19–23^, but it potentially increases the risk of intratubular uric acid crystallization especially in acidified urine, which might be harmful to the kidney^26^. It is unknown how tubular urate reabsorption and urinary uric acid excretion affect the progression of CKD. To gain a clinical insight to this issue, we examined the association of FEUA and UUCR with kidney outcome among 1042 patients with eGFR of 15‒60 mL/min/1.73 m^2^. The major finding was that both lower FEUA and UUCR were significantly associated with a higher risk of kidney failure. This association was irrespective of serum urate levels. In contrast, the highest FEUA and UUCR quartiles showed the lowest risk of kidney outcome even when urine pH was low. The results were consistent in the several sensitivity analyses and MSM. These findings suggest that inhibition of urate reabsorption might be beneficial to improve kidney outcome even though it potentially increases the risk of uric acid crystalluria.

We found that both low FEUA and UUCR were associated with kidney outcome when they decreased below certain values, which was independent of serum urate levels. Although the exact mechanism remains unknown, this result implies that urate reabsorbed in the proximal tubules, apart from hyperuricemia, might be involved in CKD progression when it exceeds a certain threshold. Several lines of evidence support a potential benefit of inhibiting urate reabsorption for the prevention of kidney injury. In vitro studies showed that inhibition of URAT1 attenuates urate-induced cell death and phenotypic transition of renal tubular cells^22,23^. In a cohort study of 874 patients with CKD who were newly prescribed urate-lowering drugs, an URAT1 inhibitor, benzbromarone, was associated with a 50%-lower risk of kidney outcomes compared to allopurinol^33^. Verinurad, a selective URAT1 inhibitor, in combination with febuxostat reduced albuminuria by approximately 50% in patients with type 2 diabetes mellitus although it is unknown whether this effect was solely attributed to the inhibition of urate reabsorption^34^. This evidence supports our finding that lower FEUA was associated with a higher risk of kidney outcome. In contrast, xanthine oxidase inhibitors, which have little effect on FEUA^35^, did not improve kidney outcome^14,15^. Future randomized controlled trials are warranted to investigate the efficacy of inhibition of urate reabsorption by uricosuric agents for hard kidney outcomes.

Despite a concern that increased urinary uric acid excretion aggravates kidney damage via promoting uric acid crystallization, we found that neither higher FEUA nor UUCR was associated with a higher risk of kidney failure even in a subgroup of patients with aciduria. Similarly, higher UUA was not associated with a higher risk of kidney failure. These findings suggest that uric acid overexcretion and high urinary uric acid concentrations do not necessarily induce clinically relevant kidney damage in the general CKD population, unlike gouty nephropathy which is characterized by precipitation of uric acid crystals in the renal tubules^36^. As we did not assess urinary uric acid crystals, our study cannot rule out the potential harmfulness of uric acid crystals. Nevertheless, we believe that the potential benefit of inhibiting urate reabsorption outweighs the possible harm arising from uric acid overexcretion. Previous studies have defined uric acid overexcretion as UUCR of > 0.5 to 0.7^37,38^, while the median [interquartile range] UUCR of the highest UUCR quartile was 0.55 [0.51–0.66] in our study. Thus, it might be possible that extremely high urinary uric acid excretion is deleterious to the kidney.

The classification of hyperuricemia has recently been redefined as “overproduction type”, “extra-renal (intestinal) underexcretion type”, and “renal underexcretion type” with certain degrees of overlap with each other^39^. The first 2 types have been regarded as “renal overloading” of urate^18^. This concept is yet incomplete because the “renal underexcretion type” can be further divided into two subtypes, “reduced glomerular filtration” and “accelerated tubular reabsorption”. Notably, we found that tubular reabsorption is a more dominant determinant of urinary uric acid excretion than glomerular filtration, as UUCR was more closely correlated with FEUA than eGFR. This may explain why uricosuric agents, such as benzbromarone, can effectively reduce serum urate levels even among patients with reduced GFR^40^. Accordingly, high UUCR does not necessarily mean “renal overloading”, but it could also arise from “diminished tubular reabsorption” which was associated with favorable kidney prognosis in our study. Conversely, low UUCR could be a result of “accelerated tubular reabsorption” which was related to poor kidney prognosis even though it has been realized as “renal underexcretion type”. We propose the measurement of FEUA in addition to UUCR as it would provide a better understanding of “renal overloading” over the classification just based on UUCR.

There are some limitations in our study. Because of the observational study design, causality between FEUA and kidney outcomes cannot be proven. Residual confounding was possible despite the extensive adjustment for measured confounders. In addition, there may be several unmeasured confounders. However, the large E-value for FEUA indicates that the possibility of unmeasured confounding that fully explains the observed association is unlikely. Since a substantial fraction of patients were excluded due to missing data on FEUA, selection bias cannot be denied although the baseline characteristics were similar between those with and without the missing data. Although we did not have data on 24-h urine samples, it is known that FEUA calculated from spot urine samples correlates well with that calculated from 24-h urine samples^41^. Importantly, while 24-h urine collection is burdensome and sometimes infeasible especially for older patients, our study revealed that FEUA measured from a spot urine sample is still valuable to predict kidney outcome. Since this was a single-center cohort study from Japan, whether our findings are applicable to different populations remains unknown.

In conclusion, lower FEUA and UUCR were associated with a higher risk of kidney failure among patients with CKD. Notably, patients in the highest quartile of FEUA and UUCR were at the lowest risk of kidney failure even among those with aciduria. Our findings suggest that accelerated urate reabsorption is harmful to the kidney. Future randomized trials are needed to elucidate the efficacy of uricosuric drugs for hard kidney outcomes.

### Supplementary Information

Supplementary Information.

## Data Availability

The dataset used in this study will be shared upon reasonable request to the corresponding author.
