# Supplementary material for: Association between urinary uric acid excretion and kidney outcome in patients with CKD
Source: Sci Rep. 2024 Mar 1;14:5119. doi: 10.1038/s41598-024-55809-9 (PMC10907602; doi:10.1038/s41598-024-55809-9)
Supplement: Supplementary file 1 — Supplementary Information. [file 41598_2024_55809_MOESM1_ESM.pdf]

Association between Urinary Uric Acid Excretion and Kidney Outcome in Patients with CKD: A Cohort Study

Yuta Asahina[1)*], Yusuke Sakaguchi[1)*], Tatsufumi Oka[1)], Koki Hattori[1)], Takayuki Kawaoka[1)], Yohei Doi[1)], Ryohei Yamamoto[1)2)], Isao Matsui[1)], Masayuki Mizui[1)], Jun-Ya Kaimori[1)] ,Yoshitaka Isaka[1)]

*YA and YS contributed equally to this work

1) Department of Nephrology, Osaka University Graduate School of Medicine, Suita, Japan
2) Health and Counseling Center, Osaka University, Toyonaka, Japan

Correspondence author:
Yusuke Sakaguchi
E-mail: sakaguchi@kid.med.osaka-u.ac.jp

Supplementary Materials

1. Supplementary methods: Statistical analysis

Marginal structural model (MSM)

2. Supplementary Figures

Figure S1. Flow diagram of the study participants

Figure S2. Subgroup analyses stratified by the presence or absence of aciduria

3. Supplementary Tables

Table S1. Comparison of baseline characteristics between those with and without data on FEUA

Table S2. Baseline characteristics according to UUCR quartile

Table S3. Competing-risk regression models for the association of FEUA and UUCR with kidney outcome

Table S4. Sensitivity analyses

Table S5. Baseline characteristics according to UUA quartile

Table S6. Additional analysis for an association between UUA and kidney outcome

Supplementary methods: Statistical analysis
Marginal structural model (MSM)

Conceptually, the MSM creates a "pseudo-population" based on inverse probability weights, where there is no association between time-dependent confounders and exposures. In the current study, we compared the risk of outcomes if all patients were continuously exposed to low FEUA (or low UUCR) with the risk of the outcome if they were never exposed to low FEUA (or low UUCR). Inverse probability weights, which are the product of the inverse probability of treatment weights and the inverse probability of censoring weights, were calculated at each 3-month follow-up period. Inverse probability of treatment weights was the reciprocal of the predicted probability of each patient having their own exposure history. This probability was estimated by fitting logistic regression models conditional on both baseline and time-dependent covariates. Similarly, inverse probability of censoring weights was the reciprocal of the probability of being uncensored as estimated by logistic regression models conditional on both baseline and time-dependent covariates. Inverse probability of treatment weights and inverse probability of censoring weights were stabilized by multiplying them by the predicted probabilities estimated by logistic regression models conditional on baseline covariates only. Inverse probability weights were truncated at the 1st and 99th percentiles.

Baseline covariates included age, sex, body mass index, systolic blood pressure, diabetes mellitus, cardiovascular comorbidities, albumin, estimated glomerular filtration rate, hemoglobin, potassium, phosphate, serum urate, C-reactive protein, urinary protein-to-creatinine ratio, loop and thiazide diuretics, angiotensin-converting enzyme inhibitors/angiotensin receptor blockers, and xanthine oxidase inhibitors. Time-dependent covariates included estimated glomerular filtration rate, phosphate, serum urate, urinary protein-to-creatinine ratio, loop and thiazide diuretics, angiotensin-converting enzyme inhibitors/angiotensin receptor blockers, and xanthine oxidase inhibitors.

We estimated hazard ratios and 95% confidence intervals by fitting inverse probability weight-weighted pooled logistic regression model, which provided estimates equivalent to those of the Cox proportional hazards model.

Figure S1. Flow diagram of the study participants

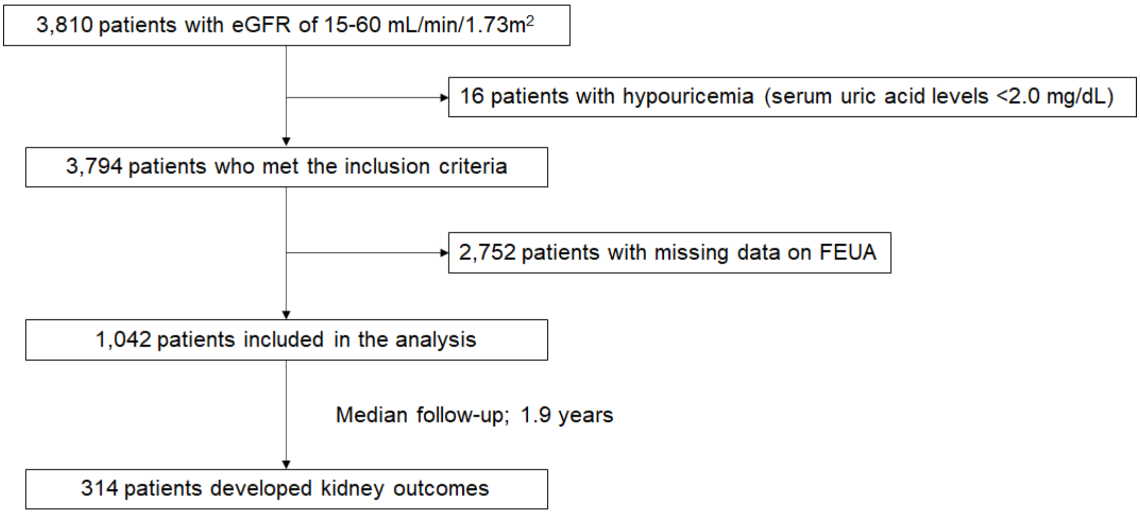

Abbreviations: eGFR, estimated glomerular filtration rate; FEUA, fractional excretion of uric acid

Figure S2. Subgroup analyses stratified by the presence or absence of aciduria

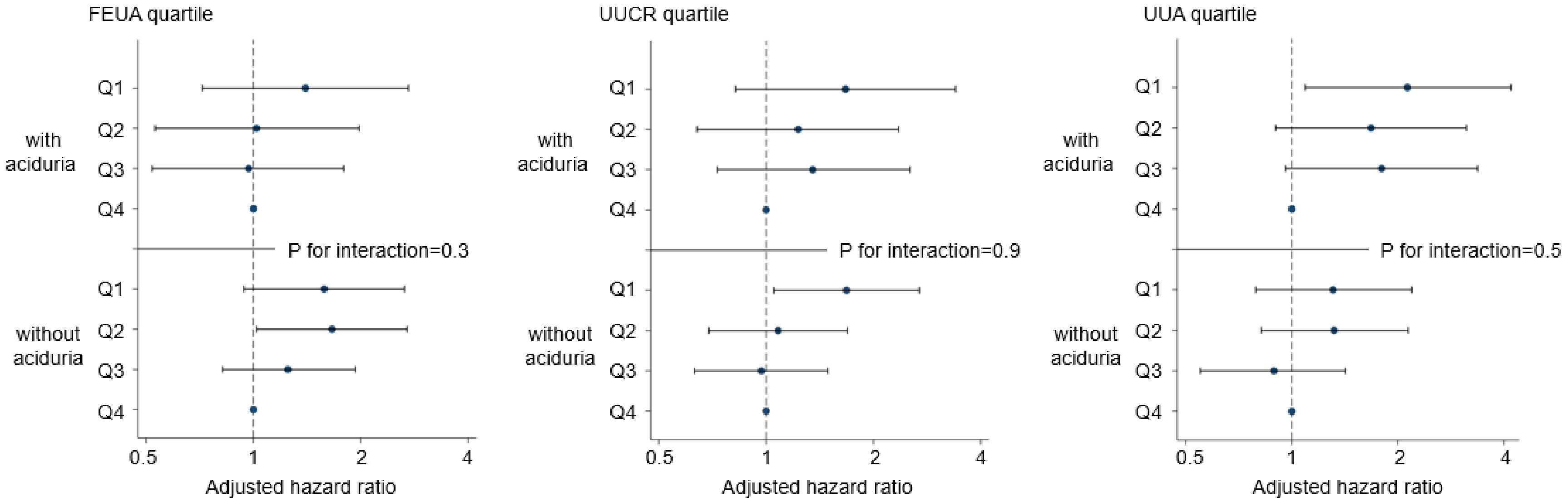

Aciduria is defined as urine pH <6.0.

Abbreviation: FEUA, fractional excretion of uric acid; UUCR, urinary uric acid-to-creatinine ratio; UUA, urinary uric acid concentrations

Table S1. Comparison of baseline characteristics between those with and without data on FEUA

Characteristics	Patients with data n=1,042	Patients without data n=2,752
Age, years	63 (16)	66 (14)
Male, %	64	65
Body mass index, kg/m^2	23.3 (4.3)	23.1 (4.0)
Systolic blood pressure, mmHg	130 (23)	131 (21)
Diastolic blood pressure, mmHg	76 (29)	75 (14)
Diabetes mellitus, %	42	33
Hemoglobin, g/dL	11.5 (2.2)	11.8 (2.2)
eGFR, mL/min/1.73 m^2	35 (12)	36 (13)
Potassium, mEq/L	4.3 (0.6)	4.4 (0.6)
Phosphate, mg/dL	3.5 (0.8)	3.5 (0.7)
Serum urate, µmol/L	411 (126)	400 (102)
Albumin, g/dL	3.4 (0.8)	3.6 (0.7)
C-reactive protein, mg/dL	0.1 [0, 1.0]	0.1 [0, 0.8]
UPCR, g/gCre	0.5 [0.1, 2.2]	0.3 [0.1, 1.4]

Data presented as mean (standard deviation), median [interquartile range], or percent.

Abbreviations: FEUA, fractional excretion of uric acid; eGFR, estimated glomerular filtration rate; UPCR, urinary protein-to-creatinine ratio

Table S2. Baseline characteristics according to UUCR quartile

Characteristics	Total n=1,042	Missing data n (%)	UUCR quartile (g/gCre) Q1: < 0.22 n=264	Q2: 0.22-0.33 n=251	Q3: 0.33-0.47 n=275	Q4: > 0.47 n=252	P for trend
Age, year	63 (16)	0	65 (16)	63 (16)	61 (16)	63 (14)	0.001
Male	667 (64%)	0	209 (79%)	179 (71%)	167 (61%)	112 (44%)	<0.001
BMI, kg/m^2	23.3 (4.3)	146 (14%)	23.9 (4.1)	23.1 (4.0)	23.5 (4.7)	22.6 (4.3)	0.001
SBP, mmHg	130 (23)	194 (19%)	128 (22)	132 (25)	130 (22)	131 (25)	0.3
DBP, mmHg	75 (15)	194 (19%)	72 (14)	75 (15)	77 (14)	75 (15)	0.003
Diabetes mellitus	433 (42%)	0	122 (46%)	106 (42%)	103 (37%)	102 (40%)	0.1
Cardiovascular comorbidities	251 (24%)	0	93 (35%)	61 (24%)	47 (17%)	50 (20%)	<0.001
Gout	84 (8%)	0	41 (16%)	21 (8%)	9 (3%)	13 (5%)	<0.001
ACEIs/ARBs	481 (46%)	0	160 (61%)	114 (45%)	109 (40%)	98 (39%)	<0.001
Loop diuretics	458 (44%)	0	157 (59%)	97 (39%)	93 (34%)	111 (44%)	<0.001
Thiazide diuretics	150 (14%)	0	64 (24%)	40 (16%)	27 (10%)	19 (8%)	<0.001
Xanthine oxidase inhibitors	345 (33%)	0	181 (69%)	87 (35%)	51 (19%)	26 (10%)	<0.001
Uricosuric agents	36 (3%)	0	10 (4%)	10 (4%)	5 (2%)	11 (4%)	0.9
Hemoglobin, g/dL	11.5 (2.2)	46 (4%)	11.7 (2.2)	11.8 (2.1)	11.6 (2.2)	10.9 (2.3)	<0.001
Potassium, mEq/L	4.3 (0.6)	11 (1%)	4.4 (0.6)	4.3 (0.6)	4.2 (0.6)	4.2 (0.6)	<0.001
Phosphate, mg/dL	3.5 (0.8)	232 (22%)	3.6 (0.8)	3.6 (0.8)	3.5 (0.7)	3.3 (0.9)	0.002
Albumin, g/dL	3.4 (0.8)	47 (4%)	3.5 (0.7)	3.5 (0.8)	3.4 (0.8)	3.2 (0.8)	<0.001
eGFR, mL/min/1.73 m^2	35 (12)	0 (0%)	30 (11)	34 (12)	38 (12)	38 (13)	<0.001
Serum urate, μmol/L	411 (126)	0 (0%)	416 (123)	452 (123)	399 (118)	378 (130)	<0.001
C-reactive protein, mg/dL	0.2 [0.0-1.0]	94 (9%)	0.2 [0.0-1.0]	0.1 [0.0-0.7]	0.1 [0.0-0.6]	0.3 [0.0-1.8]	0.2
Aciduria*	433 (42%)	4 (<1%)	130 (49%)	113 (45%)	109 (40%)	81 (33%)	<0.001
UPCR, g/gCre	0.5 [0.1-2.2]	270 (26%)	0.4 [0.1-1.5]	0.5 [0.1-2.3]	0.5 [0.1-2.7]	0.8 [0.2-2.6]	0.002
FEUA, %	7.2 [4.8-10.9]	0 (0%)	4.1 [3.1-5.5]	6.1 [4.8-7.5]	8.7 [6.5-11.9]	12.6 [9.7-18.3]	<0.001
UUCR, g/gCre	0.33 [0.22-0.47]	0 (0%)	0.16 [0.13-0.19]	0.28 [0.25-0.31]	0.40 [0.36-0.43]	0.55 [0.51-0.66]	<0.001

*Aciduria was defined as urine pH <6.0.

Abbreviations: BMI, body mass index; SBP, systolic blood pressure; DBP, diastolic blood pressure; ACEIs/ARBs, angiotensin-converting enzyme inhibitors/angiotensin II receptor blockers eGFR, estimated glomerular filtration rate; UPCR, urinary protein-to-creatinine ratio; FEUA, fractional excretion of uric acid; UUCR, urinary uric acid-to-creatinine ratio

Table S3. Competing-risk regression models for the association of FEUA and UUCR with kidney outcome

Exposures	FEUA quartile (%)				UUCR quartile (g/gCre)			
	Q1: < 4.8 (n=261)	Q2: 4.8-7.2 (n=256)	Q3: 7.2-10.9 (n=264)	Q4: > 10.9 (n=261)	Q1: < 0.22 (n=264)	Q2: 0.22-0.33 (n=251)	Q3: 0.34-0.47 (n=275)	Q4: > 0.47 (n=252)
Subhazard Ratio (95% CI)	1.70 (1.15-2.50)	1.44 (0.98-2.12)	1.31 (0.93-1.86)	ref	1.68 (1.16-2.44)	1.17 (0.82-1.67)	1.09 (0.76-1.56)	ref
P-value	0.008	0.07	0.1		0.006	0.4	0.6	

Models are adjusted for age, sex, body mass index, systolic blood pressure, diabetes mellitus, cardiovascular comorbidities, albumin, estimated glomerular filtration rate, hemoglobin, potassium, phosphate, serum urate, C-reactive protein, urinary protein-to-creatinine ratio, loop and thiazide diuretics, angiotensin-converting enzyme inhibitors/angiotensin receptor blockers, and xanthine oxidase inhibitors.

Abbreviations: FEUA, fractional excretion of uric acid; UUCR, urinary uric acid-to-creatinine ratio; CI, confidence interval

Table S4. Sensitivity analyses

1) Excluding patients followed up for <90 days

Exposures	FEUA quartile (%)				UUCR quartile (g/gCre)			
	Q1: < 4.8 (n=225)	Q2: 4.8-7.2 (n=223)	Q3: 7.2-10.9 (n=213)	Q4: > 10.9 (n=195)	Q1: < 0.22 (n=225)	Q2: 0.22-0.33 (n=211)	Q3: 0.34-0.47 (n=228)	Q4: > 0.47 (n=192)
No. of events	78	65	63	63	80	70	63	56
Incidence rate, 100 p-y (95% CI)	9.7 (7.7-12.1)	7.9 (6.2-10.1)	7.5 (5.8-9.5)	8.3 (6.5-10.7)	12.0 (9.7-15.0)	8.5 (6.7-10.8)	6.5 (5.1-8.2)	7.4 (5.7-9.6)
Hazard Ratio (95% CI)	1.68 (1.09-2.59)	1.50 (0.99-2.27)	1.08 (0.74-1.58)	ref	1.73 (1.13-2.64)	1.25 (0.85-1.85)	1.02 (0.70-1.50)	ref
P-value	0.02	0.06	0.7		0.01	0.3	0.9	

2) Excluding patients with a history of gout

Exposures	FEUA quartile (%)				UUCR quartile (g/gCre)			
	Q1: < 4.8 (n=228)	Q2: 4.8-7.2 (n=244)	Q3: 7.2-10.9 (n=242)	Q4: > 10.9 (n=254)	Q1: < 0.22 (n=223)	Q2: 0.22-0.33 (n=230)	Q3: 0.34-0.47 (n=266)	Q4: > 0.47 (n=239)
No. of events	75	70	73	71	81	66	75	65
Incidence rate, 100 p-y (95% CI)	10.9 (8.7-13.7)	8.7 (6.9-11.0)	9.3 (7.4-11.7)	9.8 (7.7-12.3)	14.8 (11.9-18.4)	9.2 (7.2-11.7)	7.5 (6.0-9.4)	8.9 (7.0-11.3)
Hazard Ratio (95% CI)	1.75 (1.15-2.65)	1.51 (1.01-2.25)	1.23 (0.87-1.74)	ref	1.82 (1.23-2.68)	1.17 (0.81-1.70)	1.07 (0.75-1.53)	ref
P-value	0.008	0.05	0.3		0.003	0.4	0.7	

Models are adjusted for age, sex, body mass index, systolic blood pressure, diabetes mellitus, cardiovascular comorbidities, albumin, estimated glomerular filtration rate, hemoglobin, potassium, phosphate, serum urate, C-reactive protein, urinary protein-to-creatinine ratio, loop and thiazide diuretics, angiotensin-converting enzyme inhibitors/angiotensin receptor blockers, and xanthine oxidase inhibitors.

Abbreviations: FEUA, fractional excretion of uric acid; UUCR, urinary uric acid-to-creatinine ratio; p-y, person-years; CI, confidence interval

Table S5. Baseline characteristics according to UUA quartile

Characteristics	Total n=1,042	Missing data n (%)	UUA quartile (mg/dL) Q1: < 0.13 n=272	Q2: 0.13-0.20 n=252	Q3: 0.21-0.31 n=276	Q4: > 0.31 n=242	P for trend
Age, year	63 (16)	0	64 (16)	64 (15)	63 (15)	60 (16)	0.002
Male	667 (64%)	0	168 (62%)	151 (60%)	187 (68%)	161 (67%)	0.1
BMI, kg/m^2	23.3 (4.3)	146 (14%)	22.9 (4.0)	23.0 (4.1)	23.6 (4.8)	23.7 (4.4)	0.03
SBP, mmHg	130 (23)	194 (19%)	126 (23)	132 (24)	131 (23)	131 (23)	0.05
DBP, mmHg	75 (15)	194 (19%)	70 (15)	75 (14)	76 (14)	78 (15)	<0.001
Diabetes mellitus	433 (42%)	0	118 (43%)	99 (39%)	134 (49%)	82 (34%)	0.2
Cardiovascular comorbidities	251 (24%)	0	95 (35%)	59 (23%)	52 (19%)	45 (19%)	<0.001
Gout	84 (8%)	0	37 (14%)	23 (9%)	12 (4%)	12 (5%)	<0.001
ACEIs/ARBs	481 (46%)	0	147 (54%)	125 (50%)	125 (45%)	84 (35%)	<0.001
Loop diuretics	458 (44%)	0	184 (68%)	119 (47%)	89 (32%)	66 (27%)	<0.001
Thiazide diuretics	150 (14%)	0	62 (23%)	42 (17%)	25 (9%)	21 (9%)	<0.001
Xanthine oxidase inhibitors	345 (33%)	0	165 (61%)	91 (36%)	58 (21%)	31 (13%)	<0.001
Uricosuric agents	36 (3%)	0	10 (4%)	14 (6%)	6 (2%)	6 (2%)	0.1
Hemoglobin, g/dL	11.5 (2.2)	46 (4%)	11.1 (2.1)	11.0 (2.1)	11.6 (2.1)	12.4 (2.4)	<0.001
Potassium, mEq/L	4.3 (0.6)	11 (1%)	4.3 (0.7)	4.2 (0.6)	4.3 (0.5)	4.3 (0.6)	0.6
Phosphate, mg/dL	3.5 (0.8)	232 (22%)	3.5 (0.9)	3.4 (0.8)	3.5 (0.7)	3.5 (0.7)	1.0
Albumin, g/dL	3.4 (0.8)	47 (4%)	3.4 (0.7)	3.4 (0.7)	3.4 (0.7)	3.5 (0.9)	0.006
eGFR, mL/min/1.73 m^2	35 (12)	0 (0%)	32 (12)	34 (11)	36 (13)	39 (12)	<0.001
Serum urate, μmol/L	411 (126)	0 (0%)	400 (127)	416 (131)	414 (124)	414 (121)	0.2
C-reactive protein, mg/dL	0.2 [0.0-1.0]	94 (9%)	0.2 [0.0-1.3]	0.1 [0.0-0.9]	0.1 [0.0-0.8]	0.1 [0.0-0.8]	0.05
Aciduria*	433 (42%)	4 (<1%)	115 (42%)	90 (36%)	111 (41%)	117 (48%)	0.1
UPCR, g/gCre	0.5 [0.1-2.2]	270 (26%)	0.3 [0.1-1.8]	0.6 [0.2-2.2]	0.6 [0.1-2.7]	0.5 [0.1-2.0]	0.07
FEUA, %	7.2 [4.8-10.9]	0 (0%)	5.1 [3.8-8.4]	7.2 [5.3-11.7]	8.2 [6.0-12.5]	8.0 [5.6-10.9]	<0.001
UUCR, g/gCre	0.33 [0.22-0.47]	0 (0%)	0.21 [0.15-0.33]	0.33 [0.23-0.48]	0.37 [0.28-0.49]	0.40 [0.31-0.51]	<0.001

*Aciduria was defined as urine pH <6.0.

Abbreviations: UUA, urinary uric acid concentrations; BMI, body mass index; SBP, systolic blood pressure; DBP, diastolic blood pressure; ACEIs/ARBs, angiotensin-converting enzyme inhibitors/angiotensin II receptor blockers eGFR, estimated glomerular filtration rate; UPCR, urinary protein-to-creatinine ratio; FEUA, fractional excretion of uric acid; UUCR, urinary uric acid-to-creatinine ratio

Table S6. Additional analysis for an association between UUA and kidney outcome

Exposure	UUA quartile (mg/dL)			
	Q1: < 0.13 (n=272)	Q2: 0.13-0.20 (n=252)	Q3: 0.21-0.31 (n=276)	Q4: > 0.31 (n=242)
No. of events	93	86	75	60
Incidence rate, 100 p-y (95% CI)	13.1 (10.7-16.1)	12.0 (9.7-14.9)	8.3 (6.6-10.3)	6.5 (5.1-8.4)
Hazard Ratio (95% CI)	1.72 (1.16-2.55)	1.50 (1.04-2.16)	1.13 (0.79-1.62)	ref
P-value	0.007	0.03	0.5	-

Models are adjusted for age, sex, body mass index, systolic blood pressure, diabetes mellitus, cardiovascular comorbidities, albumin, estimated glomerular filtration rate, hemoglobin, potassium, phosphate, serum urate, C-reactive protein, urinary protein-to-creatinine ratio, loop and thiazide diuretics, angiotensin-converting enzyme inhibitors/angiotensin receptor blockers, and xanthine oxidase inhibitors.

Abbreviations: UUA, urinary uric acid concentrations; p-y, person-years; CI, confidence interval
